# Online verification of breath‐hold reproducibility using kV‐triggered imaging for liver stereotactic body radiation therapy

**DOI:** 10.1002/acm2.14045

**Published:** 2023-05-22

**Authors:** Bingqi Guo, Kevin Stephans, Neil Woody, Alexander Antolak, Mojtaba Moazzezi, Ping Xia

**Affiliations:** ^1^ Department of Radiation Oncology Taussig Cancer Institute, Cleveland Clinic Cleveland Ohio USA

**Keywords:** breath‐hold reproducibility, liver SBRT, triggered imaging

## Abstract

**Purpose:**

To introduce a new technique for online breath‐hold verification for liver stereotactic body radiation therapy (SBRT) based on kilovoltage‐triggered imaging and liver dome positions.

**Material and Methods:**

Twenty‐five liver SBRT patients treated with deep inspiration breath‐hold were included in this IRB‐approved study. To verify the breath‐hold reproducibility during treatment, a KV‐triggered image was acquired at the beginning of each breath‐hold. The liver dome position was visually compared with the expected upper/lower liver boundaries created by expanding/contracting the liver contour 5 mm in the superior‐inferior direction. If the liver dome was within the boundaries, delivery continued; otherwise, beam was held manually, and the patient was instructed to take another breath‐hold until the liver dome fell within boundaries. The liver dome was delineated on each triggered image. The mean distance between the delineated liver dome to the projected planning liver contour was defined as liver dome position error e_dome_. The mean and maximum e_dome_ of each patient were compared between no breath‐hold verification (all triggered images) and with online breath‐hold verification (triggered images without beam‐hold).

**Results:**

Seven hundred thirteen breath‐hold triggered images from 92 fractions were analyzed. For each patient, an average of 1.5 breath‐holds (range 0–7 for all patients) resulted in beam‐hold, accounting for 5% (0–18%) of all breath‐holds; online breath‐hold verification reduced the mean e_dome_ from 3.1 mm (1.3–6.1 mm) to 2.7 mm (1.2–5.2 mm) and the maximum e_dome_ from 8.6 mm (3.0–18.0 mm) to 6.7 mm (3.0–9.0 mm). The percentage of breath‐holds with e_dome_ >5 mm was reduced from 15% (0–42%) without breath‐hold verification to 11% (0–35%) with online breath‐hold verification. online breath‐hold verification eliminated breath‐holds with e_dome_ >10 mm, which happened in 3% (0–17%) of all breath‐holds.

**Conclusion:**

It is clinically feasible to monitor the reproducibility of each breath‐hold during liver SBRT treatment using triggered images and liver dome. Online breath‐hold verification improves the treatment accuracy for liver SBRT.

## 1 INTRODUCTION

Stereotactic Body radiation therapy (SBRT) is a commonly used focal treatment for primary and metastatic liver tumors.^1^, ^2^ Liver SBRT delivers a biologically effective dose (BED) of 100 Gy or more over a few (1–5) treatment fractions.^3^ Effective SBRT requires accurate dose delivery to tumors while sparing the sensitive gastrointestinal structures surrounding the tumor.^4^ Breath‐hold techniques, assisted by spirometry devices such as the active breathing coordinator (ABC),^5^ are often utilized to reduce the treatment margin and improve localization accuracy for liver SBRT.^6^ However, ensuring the reproducibility of breath‐holds remains a challenge.^7^, ^8^, ^9^, ^10^, ^11^, ^12^, ^13^, ^14^, ^15^, ^16^

Breath‐hold reproducibility of liver has been studied using CT,^9^, ^10^ kV and MV planar images,^9^, ^11^, ^12^ fluoroscopy,^12^ and CBCT,^7^, ^14^, ^15^, ^16^ as well as non‐radiation imaging techniques.^17^, ^18^, ^19^, ^20^ Eccles et al. measured inter‐fraction and intra‐fraction reproducibility of liver position for 14 patients treated with ABC‐assisted exhale breath‐hold.^9^ Intra‐fraction breath‐hold reproducibility, measured by repeated simulation CTs, was 0.2 ± 1.5 mm in lateral, ‐0.5 ± 1.5 mm in vertical, and ‐0.9 ± 1.5 mm in longitudinal directions. Measured by AP MV planar images, inter‐fraction positional variation of liver relative to vertebral bodies was on average 3.4 mm in the longitudinal direction. Maximum longitudinal deviation of 12 mm was seen in two patients. A similar study by Lan et al. measured reproducibility of liver centroid in three consecutive CT scans with ABC‐assisted deep inspiration breath‐hold.^10^ They reported shifts of 0.7 ± 0.7 mm in lateral, 1.8 ± 2.5 mm in vertical, and 2.2 ± 2.6 mm in longitudinal directions for 44 patients. Seven percent of the shifts in the vertical direction and 11% of the shifts in the longitudinal position were more than 5 mm. Using kV CBCT, Hawkins et al. measured liver residual error after orthogonal MV setup for patients treated with ABC‐assisted exhale breath‐hold.^14^ They reported that population systematic and random setup errors were 1.9 ± 2.3 mm in lateral, 1.3 ± 3.0 mm in vertical, and 1.1 ± 2.7 mm in longitudinal directions. Liver offsets >5 mm were observed in 33% of patients, and liver offsets >10 mm and liver deformation >5 mm were observed in a minority of patients. Zhong et al. used pre‐ and post‐treatment CBCTs to evaluate the inter‐ and intra‐fraction breath‐hold variation for 24 liver patients.^7^ They reported that inter‐fraction breath‐hold variability, which was corrected by CBCT‐based alignment, was 3.2 ± 3.0 mm in lateral, 3.1 ± 3.6 mm in vertical, and 6.8 ± 6.8 mm in longitudinal directions. Intra‐fraction breath‐hold reproducibility was 0.7 ± 2.3 mm in lateral, 1.5 ± 2.5 mm in vertical, and 2.2 ± 3.6 mm in longitudinal directions. A systematic review^21^ on free‐breathing and breath‐hold liver SBRT reports inter‐fraction breath‐hold variability of 1.8 mm in lateral [95% confidence interval (CI) 1.3–2.2], 1.4 mm in vertical (95% CI 1.2–1.7), and 2.4 mm in longitudinal (95% CI 2.1–2.7), based on nine breath‐hold studies including a total of 126 patients.

To monitor the reproducibility of breath‐holds and improve the accuracy of liver SBRT, this study introduced a new online breath‐hold verification technique based on liver dome positions and kV triggered imaging^1^.^22^, ^23^ The workflow of online breath‐hold verification is presented, and its impact on the accuracy of breath‐hold liver SBRT is demonstrated.

## 2 MATERIAL AND METHODS

### 2.1 Patients

Twenty‐five liver patients treated with stereotactic body radiation therapy (SBRT) from May 2019 to February 2022 at our institution were included in this IRB‐approved study. These patients were treated with volumetric‐modulated arc therapy (VMAT) and deep inspiration breath‐hold (DIBH) using the Active Breathing Coordinator (ABC) (Elekta Inc, Stockholm, Sweden). During treatment, kV‐triggered imaging (Varian Inc, Palo Alto, California, USA) was applied to monitor the intra‐fraction breath‐hold reproducibility. Table 1 lists the patient and treatment characteristics.

**TABLE 1:** Patient and treatment characteristics.

No. of patients	25
Age	18–76
Diagnosis	Primary liver/bile duct tumors (18) Liver metastasis (7)
Tumor (GTV) volume (cc)	Mean: 40.2 Range: 1–205
Distance of tumor center of mass to liver dome (cm)	Mean: 2.0 Range: 0.6–4.4
Prescription dose (Gy)	Mean: 46.4 Range: 30–60
No. of fractions	Mean: 3.7 Range: 1–5
No. of breath‐holds per fraction	Mean: 7.8 Range: 3–14
Treatment technique	Coplanar VMAT
Respiratory motion management	DIBH with ABC
Image guidance technique	CBCT‐based alignment, and kV‐triggered imaging for online breath‐hold reproducibility monitoring

### 2.2 Workflow

#### 2.2.1 Patient screening for breath‐hold eligibility and triggered imaging

Liver SBRT patients who may benefit from breath‐hold treatments, determined by the treating physician were trained on deep inspiration breath‐hold with the ABC device before CT simulation. Only those who can tolerate the ABC device and hold breath repeatedly for more than 20 s were selected for breath‐hold treatments and the rest were treated with free‐breathing.

Patients selected for breath‐hold treatment with target volumes in superior one‐third of the liver such that a portion of the liver dome would be expected to consistently be visualized in triggered images were selected for triggered imaging during treatment.

#### 2.2.2 Simulation

For patients selected for breath‐hold liver SBRT, the BodyFix system (Elekta Inc, Stockholm, Sweden) was used for immobilization and a planning CT under DIBH was acquired with 3 mm slice thickness. To assess the reproducibility of breath‐hold, three repeated breath‐hold CT scans were acquired at simulation and CT‐CT image registrations were conducted by a physicist to measure the liver motion between the repeated breath‐hold CTs. If the liver motion exceeded 5 mm, the treating physician was notified to decide on whether to proceed with breath‐hold treatment or to change to free‐breathing treatment. For treatment planning, breath‐hold variability in simulation was incorporated by creating an internal target volume (ITV) combining the gross tumor volume (GTV) from three repeated CT scans. The planning target volume (PTV) was an additional 5 mm uniform expansion from the ITV.

#### 2.2.3 Treatment planning

As listed in Table 1, coplanar VMAT plans with 10 MV flattening filter free (FFF) beams at a maximum dose rate of 2400 MU/min were used. The high dose rate of 2400 MU/min reduced the delivery time and the number of breath‐holds per treatment. Depending on the anatomy and complexity of the plan, 1–3 partial arc beams were typically used. The VMAT SBRT plans were optimized to cover at least 95% of PTV with prescription dose without violating the tolerance of the surrounding critical structures. An “upper liver” and a “lower liver” contours were created by expanding and contracting the liver contour 5 mm in the superior‐inferior direction, respectively. These two contours were sent to the treatment machine to assist with intra‐fraction breath‐hold reproducibility monitoring.

#### 2.2.4 Treatment delivery with online breath‐hold verification

**FIGURE 1:**
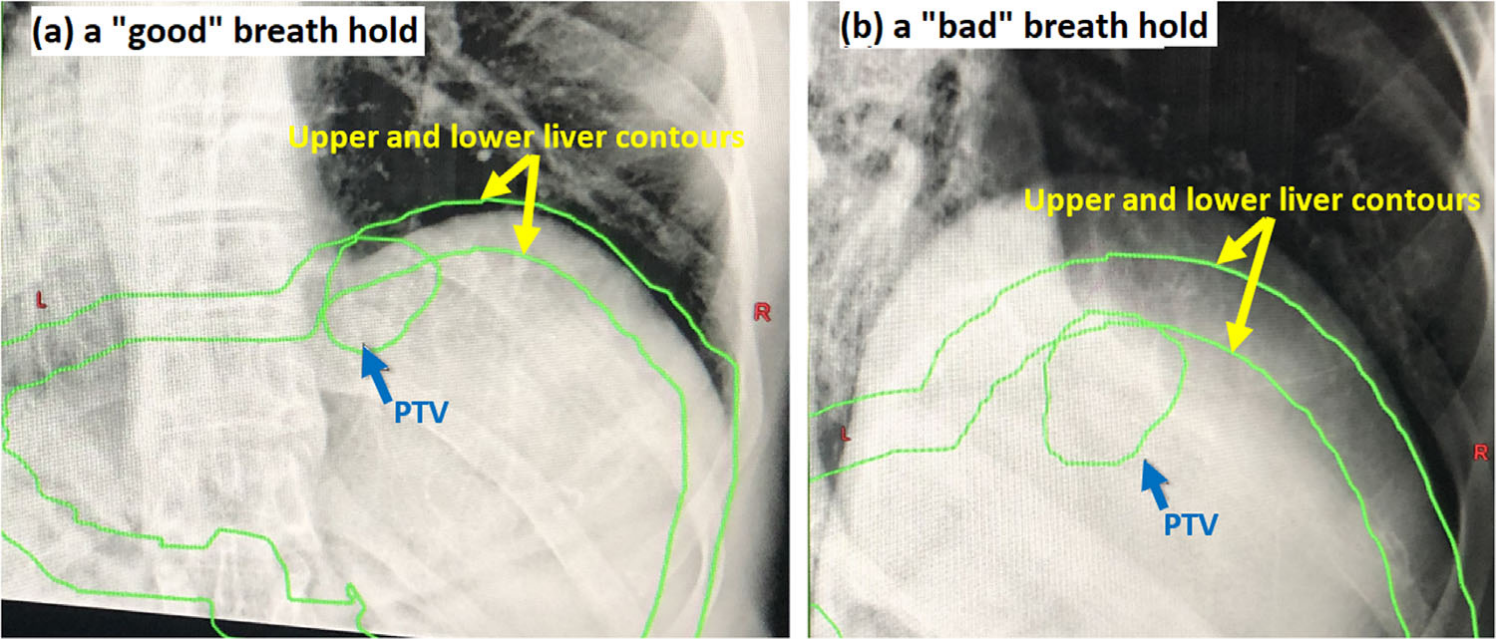
Illustration of online breath hold verification for liver SBRT. A KV triggered image was taken at the beginning of each breath hold. The liver contour was expanded/contracted 5 mm in the sup‐inf direction to create “upper” and “lower” liver boundaries, which were projected to the triggered images. If the liver dome position near PTV was outside of the boundary, the beam was held; the breath‐hold was discarded and the patient was instructed to take another breath‐hold.

kV‐CBCT acquired via 3–4 breath‐holds was used for pre‐treatment patient setup. Alignment was primarily based on liver matching of setup CBCT to planning CT. For online breath‐hold reproducibility monitoring, triggered imaging was setup pre‐treatment using the “imaging during” feature of the treatment machine and configured to take a single “triggered” kV planar image using the abdomen protocol at the beginning of each breath‐hold throughout treatment delivery. During treatment, at the beginning of each breath hold, the kV‐triggered image and the liver dome position in the image was visually compared with the upper and lower liver boundaries from planning CT. Delivery was held during kV‐triggered image acquisition and evaluation. If the liver dome position in the triggered image was within the upper and lower liver boundaries, the breath‐hold was considered a “good” breath‐hold, and delivery continued. Otherwise, the beam was manually held, the breath‐hold was discarded, and the patient was instructed to take another breath‐hold until the liver dome position was within the range. Figure 1 illustrates the technique. Typically, the first 2–3 s of each breath hold was spent on acquisition and evaluation of the triggered image.

### 2.3 Analysis of triggered images

**FIGURE 2:**
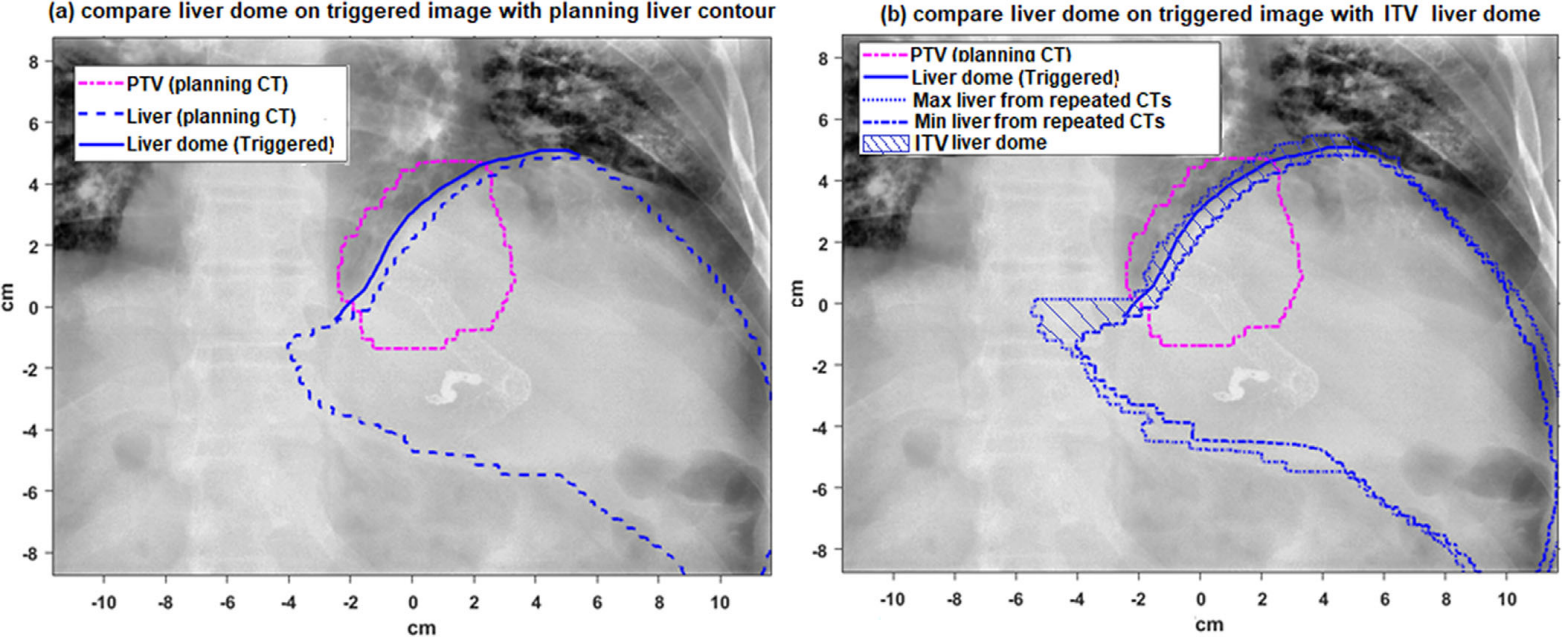
(a) The mean distance between the delineated liver dome to the projected planning liver contour was defined as liver dome position error e_dome_. (b) ITV liver dome was created by combining the liver dome from repeated breath‐hold simulation CTs and projected to the imaging plane. The mean distance between the delineated liver dome to projected ITV liver dome region was defined as e_dome_ITV_. Only liver dome within 5 cm of PTV was included for analysis.

Seven hundred thirteen breath‐hold triggered images from 92 fractions were exported from the treatment machine for analysis. After adjusting the image contrast, the liver dome was manually delineated on each triggered image and compared with the projected liver contour from planning CT. The mean distance (2D Euclidean distance) between the delineated liver dome to the projected planning liver contour was defined as liver dome position error e_dome_. Only liver dome within 5 cm of PTV was included for analysis. Figure 2a illustrates how e_dome_ was defined and calculated. e_dome_ quantifies the irreproducibility (compared with the planned position from simulation) of the breath‐holds during treatments. E_dome_ is a measurement of the targeting error at treatments due to breath‐hold irreproducibility.

For each patient, the number of triggered images (breath‐holds) that resulted in manual beam hold was counted; the mean and max e_dome_ of all triggered images (e_dome_ without breath‐hold verification) were compared with the mean and max e_dome_ of the “accepted” triggered images without beam hold (e_dome_ with online breath‐hold verification).

In addition to online breath‐hold verification, motion encompassing technique was used in planning to account for breath‐hold variability. ITV was created by combining GTVs from repeated breath‐hold simulation CTs. To analyze the effect of motion encompassing (ITV) technique on reducing targeting error, the liver was contoured on all repeated breath‐hold simulation CTs, and an ITV liver dome was created by combining the liver dome from repeated CTs, as shown in Figure 2b. The mean distance between the delineated liver dome on the triggered image to ITV liver dome (shaded region in Figure 2b) was defined as the residual liver dome error with ITV technique e_dome_ITV_. Liver dome in triggered image within the dome‐ITV implied that tumor was within the ITV. For each patient, the mean and max e_dome_ITV_ were compared with the mean and max e_dome_. Only liver dome within 5 cm of PTV was included for analysis.

### 2.4 Statistical analysis

*t*‐test was used to compare liver dome positioning errors with different breath‐hold reproducibility management techniques (no breath‐hold reproducibility management, online breath‐hold verification technique, ITV technique, and a combination of online breath‐hold verification and ITV technique), with significance defined as *p* < 0.05.

Pearson's correlation was used to evaluate the correlation between the mean and max e_dome_ of each patient with his/her age, tumor (GTV) volume, and distance of tumor centroid to the liver dome.

To evaluate whether breath‐hold variability measured at simulation is predictive of breath‐hold irreproducibility at treatment, receive operating characteristic (ROC) analysis was performed. At simulation, irreproducible breath‐hold was defined as liver dome variation from repeated breath‐hold CTs > 5 mm, and at treatment, irreproducible breath‐hold was defined as more than 5% of all breath‐holds with e_dome_ > 5 mm or max e_dome_ > 10 mm. Table 2 lists the definition of true positive, true negative, false positive, false negative for ROC analysis.

**TABLE 2:** ROC analysis to evaluate whether breath‐hold variability at simulation is predictive of breath‐hold irreproducibility at treatment.

For each patient	More than 5% of triggered images w e_dome_>5 mm or max e_dome_> 10 mm	No more than 5% triggered images w e_dome_>5 mm and max e_dome_<= 10 mm
Liver dome variation from repeated breath‐hold CTs > 5 mm	True positive (TP)	False positive (FP)
Liver dome variation from repeated breath‐hold CTs ≤ 5 mm	False negative (FN)	True negative (TN)

## 3 RESULTS

### 3.1 Feasibility of online breath‐hold verification and its impact on delivery time

Online breath‐hold verification was successfully applied to all liver SBRT patients in this study. Of the 25 patients, 13 patients had at least one breath‐hold discarded due to the liver dome position out of the boundary. For each patient, an average of 1.5 breath‐holds (range 0–7) were discarded, accounting for 5% (range 0–18%) of all breath‐holds.

Online breath‐hold verification does not affect patient setup time, it may slightly increase the delivery time if one or more breath holds were discarded. For every discarded breath‐hold, an additional 15–20 s in delivery time is needed, which includes the time for triggered image acquisition, evaluation, and recovery time before the patient's ready to take another breath‐hold. For each patient, the additional delivery time due to online breath‐hold verification was estimated to be approximately 30 s on average (range 0–2 min depending on how many breath holds were discarded). All treatment fractions were completed in the 45 min scheduled time slot for breath‐hold liver SBRTs.

### 3.2 Breath‐hold reproducibility

**FIGURE 3:**
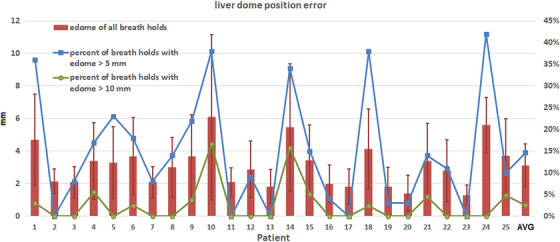
Liver dome position error for all patients. Left axis: edome of all breath‐holds for each patient; right axis: percentage of breath‐holds with edome > 5 and 10 mm, respectively.

For each patient, Figure 3 shows e_dome_ of all breath‐holds (triggered images), percentage of breath‐holds with e_dome_ > 5 mm, and percentage of breath‐holds with e_dome_ > 10 mm. For each patient, the mean e_dome_ of all breath‐holds was on average 3.1 mm (range 1.3–6.1 mm for all patients), and the maximum e_dome_ was 8.6 mm (3.0–18.0 mm). The percentage of breath‐holds with e_dome_ >5 mm was on average 15% (range 0–42% for all patients), and the percentage of breath‐holds with e_dome_ >10 mm was 3% (0–17%). Without breath‐hold verification, 15% of the breath‐holds were out of the 5 mm threshold and 3% of the breath‐holds showed large variation (> 10 mm) from planning CT. In the worst‐case scenario (patient #10), 38% of the breath‐holds exceeded the 5 mm threshold and 17% of the breath‐holds exceeded 10 mm.

To understand whether breath‐hold reproducibility correlates with patient or tumor characteristics, Pearson's correlation analysis was conducted. Pearson's correlation coefficients between mean e_dome_ and patient age, tumor (GTV) volume, and distance of tumor centroid to the liver dome were 0.10, 0.07, and 0.04, respectively. Pearson's correlation coefficients between max e_dome_ and patient age, tumor volume, and distance to the liver dome were 0.25, 0.07, and 0.24, respectively. These low correlation coefficients indicate that breath‐hold reproducibility does not depend on patient age, tumor size, or location—at least in this highly selected population. Therefore, it is necessary to evaluate breath‐hold reproducibility for each patient.

Breath‐hold variability was measured with repeated breath‐hold CTs at simulation and an action level of 5 mm was used to identify patients who may not be good candidates for breath‐hold. A question is whether breath‐hold variability measured at simulation is a good predictor for breath‐hold reproducibility at treatment. Of the 25 patients in this study, 8 showed variable breath‐hold at simulation, and 16 showed irreproducible breath‐hold at treatment. The ROC analysis reports a sensitivity of 0.38, specificity of 0.78, and accuracy of 0.59 for predicting breath‐hold irreproducibility at treatment from breath‐hold variability at simulation. The low sensitivity and high specificity indicate that while a patient showing variable breath hold in simulation is likely to show irreproducible breath hold at treatment, a reproducible breath hold at simulation does not guarantee reproducible breath‐hold at treatment. It is beneficial to monitor breath‐hold reproducibility at the time of treatment.

### 3.3 Effect of online breath‐hold verification on reducing intra‐fraction breath‐hold irreproducibility

**FIGURE 4:**
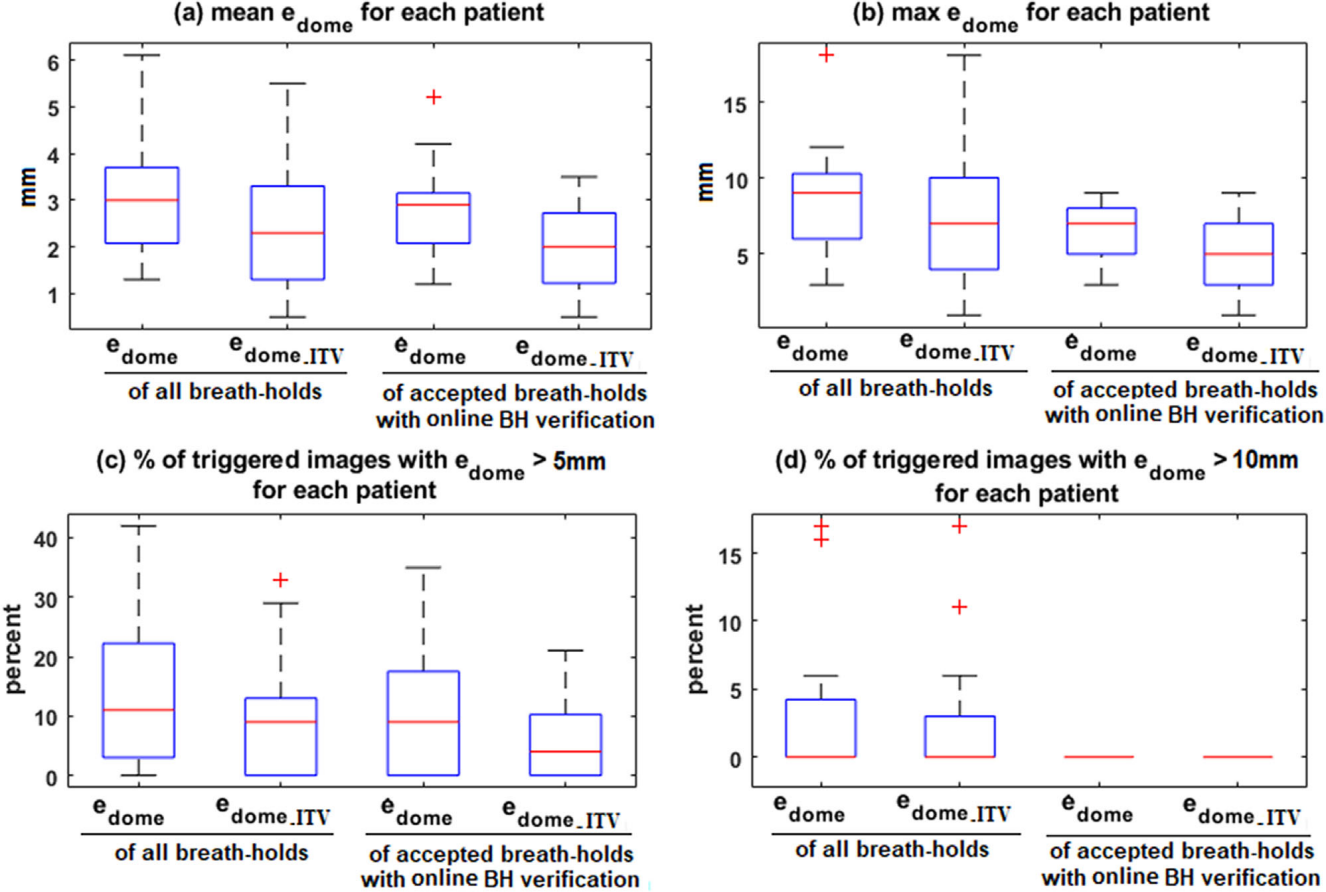
Comparison of (from left to right): liver dome position error with no breath‐hold reproducibility management technique, residual liver dome error with ITV technique, liver dome position error with online breath‐hold verification, and residual liver dome error with combination of ITV technique and online breath‐hold verification. (a) mean e_dome_/e_dome_ITV_ comparison; (b) max e_dome_/e_dome_ITV_ comparison; (c) comparison of the percentage of triggered images with e_dome_/e_dome_ITV_ > 5 mm; (d) comparison of the percentage of triggered images with e_dome_/e_dome_ITV_ > 10 mm.

Figure 4 compares the liver dome position errors with and without online breath‐hold verification. With breath‐hold verification, for each patient, the mean e_dome_ of all breath‐holds was reduced from an average of 3.1 mm (range 1.3–6.1 mm for all patients) to 2.7 mm (1.2–5.2 mm), and the maximum e_dome_ of all breath‐holds was reduced from an average of 8.6 mm (range 3.0–18.0 mm) to 6.7 mm (3.0–9.0 mm). The percentage of breath‐holds with e_dome_ >5 mm was reduced from an average of 15% (range 0–42%) to 11% (0–35%). All differences were statistically significant (*p* < 0.05). Online breath‐hold verification eliminated breath‐holds with e_dome_ > 10 mm. For the worst‐case scenario (patient #10), online breath‐hold verification reduced the mean e_dome_ from 6.1 to 4.2 mm and the max e_dome_ from 18.0 to 9.0 mm, respectively. The percentage of breath‐holds with e_dome_ >5 mm was reduced from 38% to 25%, and the percentage of breath‐holds with e_dome_ > 10 mm was reduced from 17% to 0%.

### 3.4 Effect of ITV technique on reducing targeting error caused by intra‐fraction breath‐hold irreproducibility

Figure 4 compares the liver dome position error e_dome_ with the residual liver dome error with ITV technique e_dome_ITV_. For each patient, the mean e_dome_ of all breath‐holds was on average 3.1 mm (range 1.3–6.1 mm for all patients), and the mean e_dome_ITV_ was 2.3 mm (1.0–5.5 mm). The maximum e_dome_ of all breath‐holds was on average 8.6 mm (range 3.0–18.0 mm for all patients), and the maximum e_dome_ITV_ was 7.5 mm (1.0–18.0 mm). On average 15% (range 1–42%) of the breath‐holds had e_dome_ >5 mm and 9% (0–33%) of the breath‐holds had e_dome_ITV_ >5 mm. The percentage of breath‐holds with e_dome_ >10 mm was on average 3% (range 0–17%), and the percentage of breath‐hold with e_dome_ITV_ > 10 mm was 2% (0–17%). All differences were statistically significant (*p* < 0.05). For the worst‐case scenario (patient #10), mean e_dome_ was 6.1 mm, and mean e_dome_ITV_ was 5.5 mm. Twenty‐nine percent of breath‐holds had e_dome_ITV_ >5 mm, as compared to 38% of breath holds with e_dome_ >5 mm The max e_dome_ was the same as max e_dome_ITV_, and same percentage of breath‐holds had e_dome_>10 mm vs e_dome_ITV_ > 10 mm. While ITV technique reduces targeting error caused by breath‐hold irreproducibility, its downside is an enlarged target volume thus irradiation of more normal tissue.

### 3.5 Online breath‐hold verification versus ITV technique

ITV technique was more effective than online breath‐hold verification in reducing the mean liver dome targeting error (*p* = 0.029). Two techniques were comparable in reducing the max liver dome targeting error (*p* = 0.284) and percentage of breath‐holds exceeding the 5 mm threshold (*p* = 0.335). A main advantage of the online breath‐hold verification technique is it eliminated the breath‐holds with large deviation (e_dome_ > 10 mm) where ITV method was not as effective (*p* = 0.020). Combining both techniques, for each patient, the mean e_dome_ITV_ was on average 2.0 mm (range 0.5–3.5 mm for all patients), and the maximum e_dome_ITV_ was on average 5.4 mm (range 1.0–9.0 mm). The percentage of breath‐holds with e_dome_ITV_ >5 mm was on average 6% (range 0–21%), and the percentage of breath‐holds with e_dome_ITV_ >10 mm was eliminated. Combination of two techniques yield better results than either one alone in reducing the chance of missing target due to breath‐hold irreproducibility.

## 4 DISCUSSION

Breath‐hold reproducibility is essential for liver SBRT because of the high dose gradient and close proximity of sensitive gastrointestinal structures. Using liver or liver dome as surrogates for liver tumors, previous studies have measured the breath‐hold reproducibility using various imaging modalities pre‐ and during treatments.^7^, ^8^, ^9^, ^10^, ^11^, ^12^, ^13^, ^14^, ^15^, ^16^ The systemic and random errors (3D vector) caused by breath‐hold variability/irreproducibility ranged from 1.0 to 8.1 mm, and 2.6 to 8.3 mm, respectively. The percentage of breath‐holds exceeding the 5 mm threshold ranged from 7% to 33%.^7^, ^8^, ^9^, ^10^, ^11^, ^12^, ^13^, ^14^, ^15^, ^16^ Our study reported a mean breath‐hold irreproducibility (between treatment and simulation) of 3.1 mm and 15% of breath‐holds exceeding 5 mm threshold, in agreement with previous publications. Our result along with previous studies showed that a non‐negligible percentage of breath‐holds may exceed the 5 mm threshold although the mean breath‐hold irreproducibility was small (less than 5 mm). For some patients, the maximum breath‐hold irreproducibility may be greater than 10 mm (18 mm maximum in this study), and the percentage of breath‐holds exceeding the 5 mm threshold may be significant (42% maximum in this study). Therefore, it is necessary to monitor the reproducibility of breath‐holds for liver SBRT patients. Moreover, our results showed that breath‐hold reproducibility does not depend on the patient or tumor characteristics. Measuring breath‐hold variability at simulation can identify some patients with irreproducible breath‐hold pattern and generate ITV to reduce targeting errors due to breath‐hold irreproducibility. However, the low sensitivity of prediction warns us that for patients who showed reproducible breath‐hold at simulation, there is still a probability their breath‐hold may be irreproducible at treatment. Therefore, it is important to verify the reproducibility of each breath‐hold at the time of treatment for every patient.

This is the first clinical study to verify the reproducibility of each breath‐hold at the time of treatment for liver SBRT patients treated with linear accelerators using triggered imaging to the liver dome. Using triggered images and manual beam hold to filter out irreproducible breath‐holds improved the accuracy of breath‐hold liver SBRT with minimal addition to the clinical workflow: adding triggered imaging in delivery.

In this study, the liver dome was used as the surrogate for liver tumors, similar to previous publications.^7^, ^8^, ^9^, ^10^, ^11^, ^12^, ^13^, ^14^, ^15^, ^16^ However, a questions is whether the liver dome is a good surrogate for liver tumors. A study published by Kawahara et al. compared the shifts based on liver dome matching of CBCT with reference CT with the gold standard of matching lipiodol uptake after transarterial chemoembolization for 59 liver SBRT patients.^15^ They concluded that the error of liver dome match was 0.6 ± 0.8 mm in lateral, 1.0 ± 1.4 mm in vertical, and 1.2 ± 1.3 mm in longitudinal directions, significantly lower than bone‐based match. Another study by Seppenwoolde et al. concluded that while fiducial markers in or near the tumor were the best surrogates for the tumor, the 3D position of the diaphragm dome was the second‐best predictor with an error of 1.2 ± 1.6 mm in lateral, 2.1 ± 1.5 mm in vertical, and 2.3 ± 1.6 mm in longitudinal directions.^24^ In this study, only one patient (patient #15) had a marker implanted in the tumor that is visible in the triggered images. For this patient, the 3D positioning error of the marker centroid was 3.2 ± 1.5 mm in 39 triggered images, close to the liver dome position error of 3.4 ± 2.2 mm.

Using radiographic imaging to monitor the tumor motion during treatment may raise concerns about extra imaging dose to the patient. In this study, on average 7.75 triggered images were taken per treatment fraction. The dose of a typical triggered image (100 kVp, 200 mA, 65 ms) is 0.125 cGy. Additional imaging dose was on average 0.97 cGy per fraction, less than the dose of an abdominal CBCT.

A limitation of the online breath‐hold verification technique is that, by acquiring a kV‐triggered imaging at the beginning of each breath‐hold, it accounts for the reproducibility between different breath‐holds but does not account for the stability within each breath‐hold. While most previous studies, such as publications by Eccles et al.^9^ and Dawson et al.,^12^ showed minimal or no motion within breath‐holds, a recent publication by Farrugia et al. found breath‐hold stability to be of the same magnitude as the breath‐hold reproducibility.^25^ For deep inspiration breath‐hold, the breath‐hold stability was 2.5 ± 1.9 mm, while the reproducibility was 2.7 ± 2.5 mm. A possible solution to account for breath‐hold stability may be to use fluoroscopy imaging instead of a single kV‐triggered image, at the expense of a higher imaging dose to the patient. Non‐radiation imaging, such as surface imaging,^17^ ultrasound,^18^ MR‐guided radiation therapy,^19^, ^20^ or implanted electromagnetic transponders^23^ may be alternatives to monitor the breath‐hold reproducibility and stability.

Another limitation of the online breath‐hold verification technique is that it relies on manual beam hold based on treatment staff's judgment on whether the liver dome position in the triggered images was within the defined boundaries. The visibility of the liver dome may depend on the patient's anatomy, the location of the tumor/isocenter, and the x‐ray angles of the triggered images. In this study, due to the careful patient selection (all patients included had tumors in the upper liver and clear liver/lung interphase as seen in the simulation CT), the liver dome (or at least part of the liver dome) was visible in all triggered images. The results of this study showed that visual examination of liver dome position and manual beam hold is very effective in eliminating large (>10 mm) deviations and reducing the percentage of moderate deviations (5–10 mm). However, for liver SBRT, accuracy of less than 5 mm is desirable. To improve the treatment accuracy, automatic delineation of the liver dome/diaphragm^26^, ^27^ and automatic beam gating is necessary and will be a future direction of this study.

The online breath‐hold verification technique requires the liver dome to be present in all triggered images, which limits its application to tumors in the upper liver. On the other hand, tumors in the upper liver tend to move more in breathing and thus are more likely to be selected for breath‐hold treatment. A breath‐hold verification technique that aims to improve breath‐hold reproducibility can be beneficial to this group of patients. Additionally, patients with fiducials, TIPS (Transhepatic‐Inter Caval – Porto – Systemic Shunt), lipiodol, or other material visible in kV planar images can also be considered for online breath‐hold verification using triggered imaging, in addition to patients whose tumors are in the upper liver.

## 5 CONCLUSION

It is clinically feasible to monitor the reproducibility of each breath‐hold during liver SBRT treatment using kV‐triggered images and liver dome positions. The treatment accuracy for breath‐hold liver SBRT is improved with online breath‐hold verification.

## AUTHOR CONTRIBUTIONS

Bingqi Guo, Kevin Stephans, and Ping Xia contributed to all aspects of the research, including study design, data acquisition, data analysis, drafting the manuscript, and manuscript revision. Neil Woody, Alexander Antolak, and Mojtaba Moazzezi contributed to data acquisition, data analysis, and manuscript revision.

## CONFLICT OF INTEREST STATEMENT

Dr. Xia reports grants from Philips Medical Solution, grants from AVO, outside the submitted work.

## Footnotes

1 Triggered imaging is a capability of the Varian TrueBeam delivery system that acquires single kV images during treatment delivery.
